# P-2046. Unnecessary Urine Culture Testing and Treatment of Asymptomatic Bacteriuria in Hospitalized Non-Catheterized Patients: Finding the Right Target for Diagnostic Stewardship Interventions

**DOI:** 10.1093/ofid/ofaf695.2210

**Published:** 2026-01-11

**Authors:** Sergio Reyes Salcedo, Kathleen Degnan, Maiko Suarez, Lauren Dutcher

**Affiliations:** University of Pennsylvania Perelman School of Medicine, Swanzey, NH; University of Pennsylvania Perelman School of Medicine, Swanzey, NH; University of Pennsylvania Perelman School of Medicine, Swanzey, NH; University of Pennsylvania Perelman School of Medicine, Swanzey, NH

## Abstract

**Background:**

Asymptomatic bacteriuria (ASB) is common in hospitalized patients. With just a few exceptions, screening and treatment for ASB is not recommended. However, many of these patients receive antibiotics inappropriately. The aim of this study was to determine the prevalence of ASB and inappropriate treatment in non-catheterized patients in the inpatient setting. This is a population that has not been specifically addressed in previous studies and may represent an important proportion of patients with ASB.

**Table 1.**
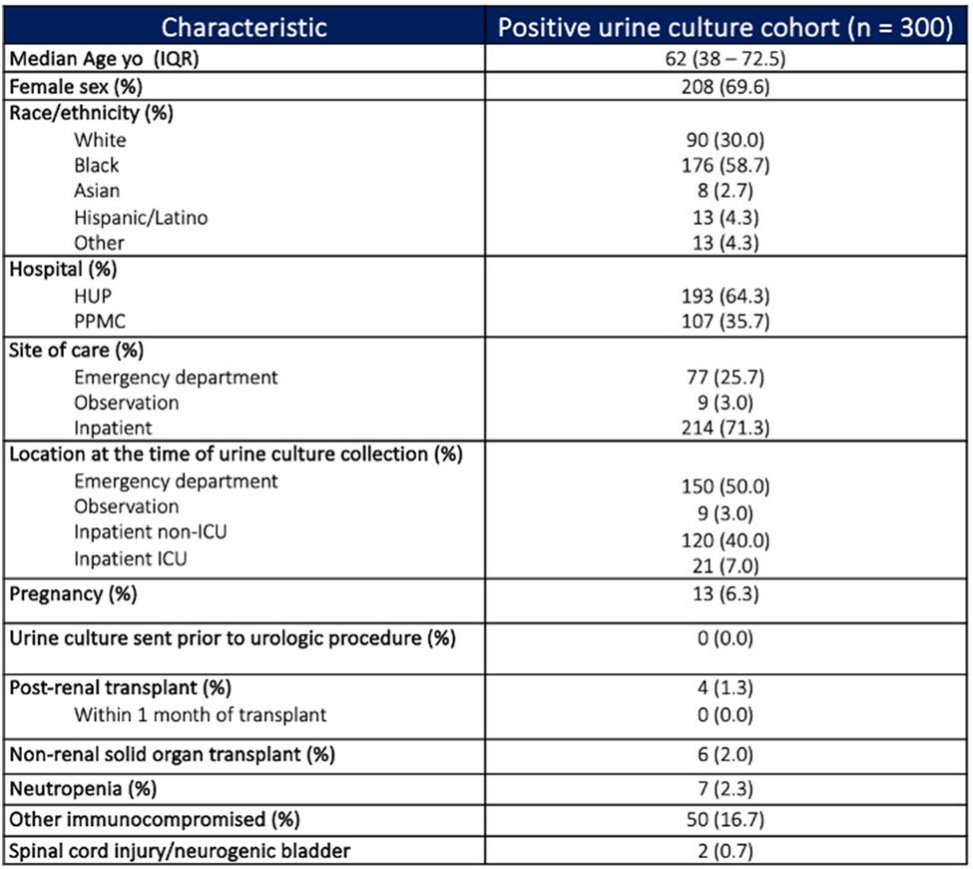
Demographics and Other Characteristics of the Positive Urine Culture Cohort

**Figure 1.**
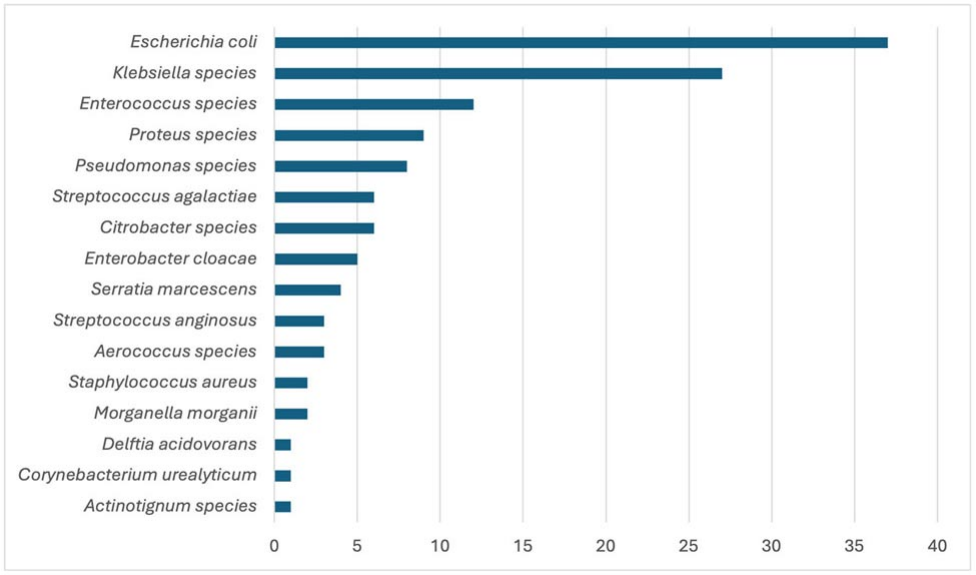
Microorganisms Identified in Urine Cultures (n=127*) *This is the total number of non-contaminated organisms. Some patients had more than one organism growing in the urine culture

**Methods:**

We randomly selected a sample of all positive urine cultures (UC) collected inpatient at 2 academic tertiary-care hospitals between July 2023 and June 2024. We retrospectively reviewed patients' charts, excluding UC with yeast and those obtained from any type of urinary catheter. Notable variables collected included: location of the patient at the time of UC collection (i.e., ED, Observation unit, wards, and ICU), bacteria isolated, and symptom documentation. We calculated the proportion of patients with ASB and those who were inappropriately treated, and median duration of antibiotic for ASB.

**Results:**

A total of 300 patients with positive UC were included. The median age was 62 years old and 208 were women (69.6%) as shown in Table 1. Of note, 64.3% of total UC represented contamination, most frequently reported as mixed flora (no significant difference was noted between the two hospitals or patient location). Frequency of organisms isolated among non-contaminated samples is shown in Figure 1. Excluding contaminated UC, 61.7% (66/107) were considered to have ASB, with 56.1% of these (37/66) inappropriately receiving antibiotics with a median duration of 5 days. In contrast, 8.3% (13/157) of contaminated UC from asymptomatic patients received antibiotics. Additionally, 71.3% of all UC were assessed to be not indicated.

**Conclusion:**

In addition to a high proportion of unnecessary UC testing and treatment of ASB in non-catheterized patients, there was an unexpectedly high rate of contaminated UC which may indicate a need for improved UC collection practices. Our results highlight the importance of evaluating UC testing practices in specific populations to develop targeted diagnostic stewardship interventions.

**Disclosures:**

All Authors: No reported disclosures

